# Rapid plant-to-plant systemic signaling via a *Cuscuta* bridge

**DOI:** 10.1093/plphys/kiae339

**Published:** 2024-06-18

**Authors:** Yosef Fichman, María Ángeles Peláez-Vico, Asha Kaluwella Mudalige, Hyun-Oh Lee, Ron Mittler, So-Yon Park

**Affiliations:** School of Plant Sciences and Food Security, George S. Wise Faculty of Life Sciences, Tel Aviv University, Tel Aviv 6997801, Israel; Division of Plant Science and Technology, College of Agriculture Food and Natural Resources and Interdisciplinary Plant Group, University of Missouri, Columbia, MO 65211, USA; Division of Plant Science and Technology, College of Agriculture Food and Natural Resources and Interdisciplinary Plant Group, University of Missouri, Columbia, MO 65211, USA; Division of Plant Science and Technology, College of Agriculture Food and Natural Resources and Interdisciplinary Plant Group, University of Missouri, Columbia, MO 65211, USA; Division of Plant Science and Technology, College of Agriculture Food and Natural Resources and Interdisciplinary Plant Group, University of Missouri, Columbia, MO 65211, USA; Department of Surgery, University of Missouri School of Medicine, Christopher S. Bond Life Sciences Center, University of Missouri, Columbia, MO 65201, USA; Division of Plant Science and Technology, College of Agriculture Food and Natural Resources and Interdisciplinary Plant Group, University of Missouri, Columbia, MO 65211, USA

## Abstract

Two plants connected via a *Cuscuta* bridge exchange rapid systemic calcium, electric, and reactive oxygen species signals, suggesting that *Cuscuta* may have beneficial effects to host plants.

Dear Editor,

The ability of plants to rapidly transmit signals from one tissue (e.g. a leaf) to another (e.g. another leaf, roots, and/or reproductive tissues), termed “systemic signaling”, plays a key role in optimizing the plant overall photosynthetic activity, growth, productivity, and responses to abiotic and biotic stresses (Kollist et al. 2019). Among the different signals that mediate rapid systemic intra-plant tissue-to-tissue communication are electric, calcium, reactive oxygen species (ROS), and hydraulic waves (e.g. Miller et al. 2009; Mousavi et al. 2013; Toyota et al. 2018; Grenzi et al. 2023). These travel at rates of 0.5-to-several cm per minute, mostly via the plant vascular system, and carry information that triggers transcriptomic, metabolomic, proteomic, and physiological responses in systemic tissues (e.g. Suzuki et al. 2013; Nguyen et al. 2018; Zandalinas et al. 2020).

In recent years, it was found that systemic signals can also travel from plant-to-plant (inter-plant), below or above ground, and convey important information that coordinates the response of different plants living in a community to stress (e.g. Venkateshwaran et al. 2013; Szechyńska-Hebda et al. 2022). However, the potential of parasitic plants, which are generally considered to be pests, to mediate rapid plant-to-plant communication in response to stress is largely unknown. Parasitic plants such as dodder (*Cuscuta campestris*) are thought to “steal” water and nutrients from host plants after forming physical connections with them, called haustoria, without providing any benefits back to the plant (Hibberd and Dieter Jeschke 2001). Over the past decade, different studies revealed the transfer of mRNAs, small RNAs, DNA, and proteins between the host and *Cuscuta* (Jhu and Sinha 2022). Mobility of mRNAs and proteins between 2 different plants connected by *Cuscuta* has also been shown (Liu et al. 2020), as well as the transfer of macromolecules associated with systemic herbivory (Hettenhausen et al. 2017; Zhuang et al. 2018). However, the potential of *Cuscuta* to transmit important rapid systemic signals, such as calcium, ROS, and membrane potential depolarization waves, between different plants in response to abiotic stress remains uncertain, prompting us to investigate the transmission of such inter-plant signals between 2 different host plants connected via a *Cuscuta* (that functions as a “bridge”).

To establish an experimental system to study the transfer of rapid systemic signals between 2 different plants connected by a *Cuscuta* bridge, we produced an Arabidopsis (*Arabidopsis thaliana*) “donor”—*Cuscuta* “bridge”—and “receiver” *Arabidopsis*, inter-plant (plant-*Cuscuta*-plant) system (Fig. 1A) and studied the propagation of the ROS wave in this system. Both donor and receiver *Arabidopsis* were connected to *Cuscuta* through fully developed haustoria (Fig. 1B, black arrow). Using a live whole-plant imaging system (IVIS Lumina S5; Fichman et al. 2022), we then tracked the spread of the ROS wave within the inter-plant system after wounding a single rosette leaf of the donor *Arabidopsis* plant (Fig. 1A, yellow lightning bolt; this treatment was previously shown to send a systemic signal to neighboring leaves connected via the vascular system, and to the entire plant; Fichman et al 2022). Interestingly, the accumulation of the systemic ROS signal was not limited to the local leaf of the donor plant but rather spread to and throughout the donor systemic leaves, *Cuscuta* bridge, and the entire receiver plant (Fig. 1C). This finding suggested that wounding of a single leaf of the donor plant triggered a systemic ROS wave that traveled through the *Cuscuta* bridge and triggered ROS accumulation in the receiver plant.

**Figure 1.**
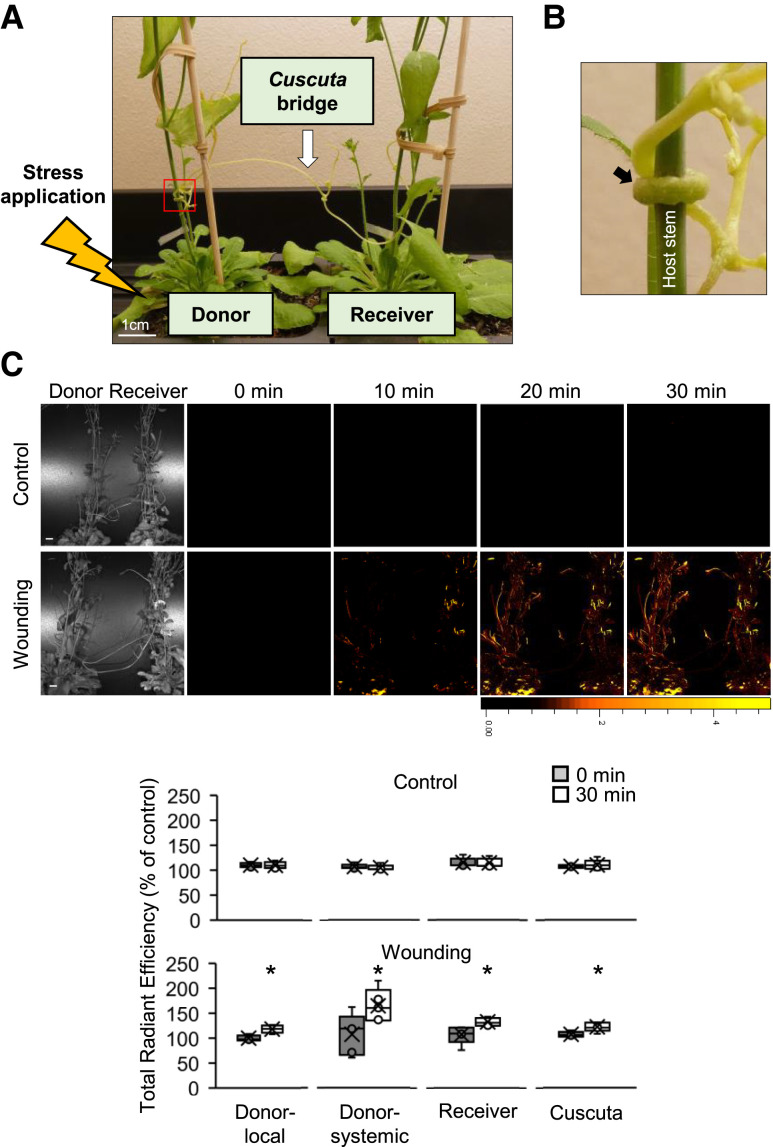
The experimental design used for measuring ROS accumulation following wounding. **A)** A *Cuscuta* bridge (arrow) connected 2 *Arabidopsis* plants (donor *Arabidopsis* on the left and receiver *Arabidopsis* on the right); stress was applied to a single leaf of the donor plant (jagged arrow). **B)** Enlarged image of the red box in **A)**. The arrow indicates established haustorium developed on the *Arabidopsis* stem. **C)** Representative 30 min time lapse images of ROS accumulation in control plants or following mechanical wounding stress applied to a single leaf of the donor plant (top), and quantification of fluorescence corresponding to ROS content at 0 min (gray) and after 30 min (white) in control treatment and following an injury of a single leaf of the donor plants, in donor's local and systemic leaves, and in the receiver plant and *Cuscuta* bridge (bottom). Asterisks indicated significance; Student's *t*-test (*N* = 5; *P < 0.05). Results are displayed as box-and-whisker plots, with the borders corresponding to the 25th and 75th percentiles of the data. Each data value is included as a point within each box plot, with the horizontal line representing the median and “X” corresponding to the mean. Whiskers represent 1.5 times the minimum and maximum of the mean (1.5 times of the interquartile range); the size bar indicates 1 cm and is applicable to all other fluorescence images; units of color scale bar are total counts of fluorescence; fluorescence units used to calculate % of control are (p/s]/[µW/cm^2^; Fichman and Mittler 2021).

Using the system developed in Fig. 1, we next examined the propagation of other rapid systemic signals from plant-to-plant through the *Cuscuta* bridge. To prevent potential interference from ROS signaling processes associated with the penetration of the *Cuscuta* haustoria into the host stems (which is a type of wounding stress; Johnsen et al. 2015; Hegenauer et al. 2016; Slaby et al. 2021), we used high light stress as a trigger of the different rapid systemic waves (Fig. 2). Applying high light stress to a single rosette leaf of a donor *Arabidopsis* plant led to ROS accumulation within 30 min in the entire plant-*Cuscuta*-plant chain including the donor, *Cuscuta*, and receiver plants (Fig. 2A; Video 1; representative of 5 different experiments; in all videos, color scale shows increase and decrease of the signal; zero is black; Fichman et al. 2022). In contrast, when *Cuscuta*-inoculated donor and *Cuscuta*-inoculated receiver plants were not connected via a *Cuscuta* bridge, the ROS signal did not cross over from the donor to the receiver plant, indicating that, at least under the conditions tested, volatile signals were not involved in the transmission of the plant-to-plant signal (Supplementary Fig. S1; for this analysis, we also directly compared the intensity of signals between receiver plants in Fig. 2A and Supplementary Fig. S1A; Supplementary Fig. S1C). As the ROS wave is frequently accompanied by the propagation of systemic calcium and electric waves, we repeated our experiments using the same plant-*Cuscuta*-plant chain system, however, using dye indicators for calcium and membrane potential (electric) waves (Fichman and Mittler 2021; Fichman et al. 2022). Similar to the ROS wave observation (Fig. 2A; Video 1), we were able to image and measure the propagation of a calcium wave (Fig. 2B; Video 2; representative of 5 different experiments), and a membrane depolarization wave (Fig. 2C; Video 3; representative of 5 different experiments), across the entire plant-*Cuscuta*-plant system, in response to the local application of high light stress to a single leaf of the donor plant. Please note that soil bacteria/algae, as well as dead leaves, can produce a fluorescence signal that would be visible in Videos 1 to 3. These are not associated with the plant-to-plant signal and should be considered as background signals. Taken together, our findings reveal that ROS, calcium, and membrane potential waves, activated by a high light stress treatment applied to a single tissue (leaf) of the donor plant, are transmitted to a receiver plant through the *Cuscuta* bridge. These results indicate that systemic stress signals (ROS, calcium, and electric waves) can be transferred within minutes aboveground between different plants connected through a parasitic plant such as *Cuscuta*.

**Figure 2.**
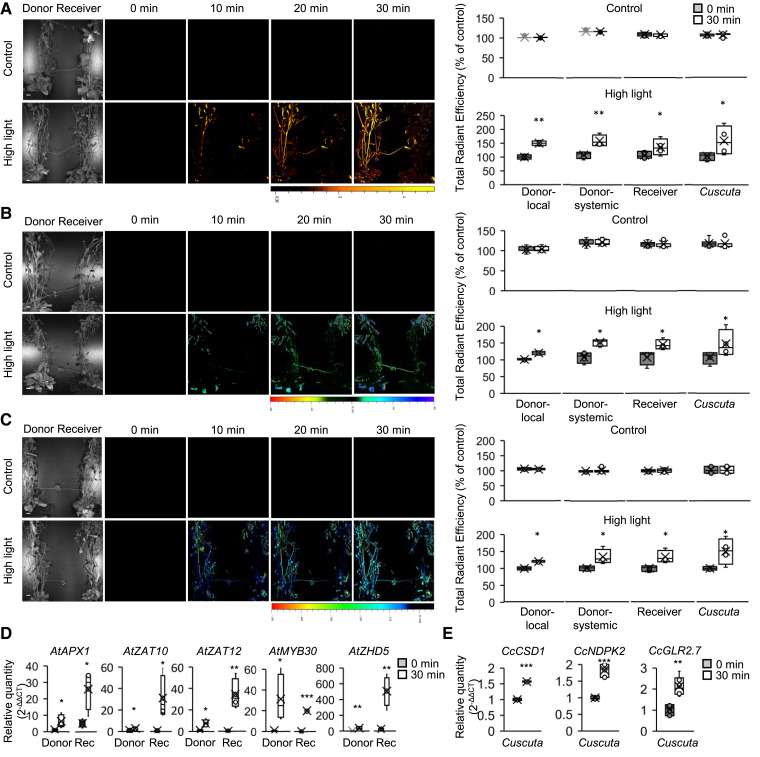
ROS accumulation and transcriptional changes in donor plant, *Cuscuta* bridge, and receiver plant, in response to a local light stress applied to a single leaf of the donor plant. **A)** Representative images of ROS accumulation in control and high light stress-treated donor plant, in a donor-*Cuscuta* bridge-receiver chain (light stress was applied to a single leaf of the donor plant; left), and quantification of fluorescence corresponding to ROS content at 0 min (gray) and after 30 min (white) in control and following high light treatment applied to a single leaf of the donor plants (local leaf), in *Arabidopsis* donor's local and systemic leaves, receiver and *Cuscuta* plants (right). **B)** Similar to **A)** but for Ca^2+^ accumulation. **C)** Similar to **A)** but for membrane potential changes. **D)** Changes in the steady-state level of transcripts associated with ROS responses in *Arabidopsis* donor and receiver (Rec) plants, measured under control conditions (0 min; gray) or following treatment (30 min after light stress to a single leaf of the donor plant; white). **E)** Same as **D)** but for *Cuscuta* ROS-response transcripts. Results are shown as box-and-whisker plots, with the borders corresponding to the 25th and 75th percentiles of the data. Each data value is included as a point within each box plot, with the horizontal line representing the median and “X” corresponding to the mean. Whiskers represent 1.5 times the minimum and maximum of the mean (1.5 times of the interquartile range). Quantitative real-time results are presented as relative quantity PCR, normalized to reference gene. *N* = 5 to 12, asterisks indicated significant difference; Student's *t*-test (*N* = 7, 5, and 5, in **A** to **C**), respectively; *P < 0.05, **P < 0.01, and ***P < 0.001); the size bar indicates 1 cm and is applicable to other fluorescence images; units of color scale are total counts of fluorescence; fluorescence units used to calculate % of control are (p/s]/[µW/cm^2^; Fichman and Mittler 2021). *APX1*, *ASCORBATE PEROXIDASE 1*; *CSD1*, *COPPER/ZINC SUPEROXIDE DISMUTASE 1*; *GLR2.7*, *GLUTAMATE RECEPTOR 2.7*; *MYB30*, *MYELOBLASTOSIS DOMAIN PROTEIN 30*; *NDPK2*, *NUCLEOSIDE DIPHOSPHATE KINASE 2*; Rec, receiver; *ZAT10*, *ZINC FINGER OF ARABIDOPSIS THALIANA 10*; *ZAT12*, *ZINC FINGER OF ARABIDOPSIS THALIANA 12*; *ZHD5*, *ZINC FINGER HOMEODOMAIN 5.*

To investigate whether the transmission of the different waves is associated with the triggering of molecular responses in the receiver plant, we performed RT-qPCR using total RNA extracted from the different *Arabidopsis* and *Cuscuta* plants (Fig. 1A). Tissues were harvested before and after the application of the high light stress to the local tissue, and transcript levels were compared relative to the control condition. The RT-qPCR results revealed an increase in the expression of *ASCORBATE PEROXIDASE 1* (*AtAPX1*), *MYELOBLASTOSIS DOMAIN PROTEIN 30* (*AtMYB30*), *ZINC FINGER OF ARABIDOPSIS THALIANA 10* (*AtZAT10*), and *ZINC FINGER HOMEODOMAIN 5* (*AtZHD5*) transcripts in both donor and receiver *Arabidopsis* plants under the high light stress (Fig. 2D). These transcripts are known to accumulate in local and systemic tissues of *Arabidopsis* in response to a 2 min local light stress (Fichman et al. 2022). The enhanced transcript expression in receiver plants suggests that the propagation of systemic signals through the *Cuscuta* bridge stimulates a molecular response in them. Furthermore, we investigated changes in the steady-state levels of the stress-associated *Cuscuta* transcripts, *COPPER/ZINC SUPEROXIDE DISMUTASE 1* (*CcCSD1*), *NUCLEOSIDE DIPHOSPHATE KINASE 2* (*CcNDPK2*), and *GLUTAMATE RECEPTOR 2.*7 (*CcGLR2.7*), to understand the potential influence of the systemic waves on *Cuscuta*. Our RT-qPCR result revealed significant changes in the expression of these transcripts (Fig. 2E), indicating that the transmitted systemic signals from the host plant could also trigger transcriptional responses in *Cuscuta*.

When viewing the data shown in Figs. 1C and 2, A to C, it is important to note that different plant tissues can absorb the different imaging dyes, and/or respond to/metabolize ROS, calcium, and/or membrane potential at different rates, resulting in differential intensities and rates of signal imaging/propagation. For example, seed pods might absorb the dye much faster, and/or display much higher rates of ROS production/much lower rates of ROS scavenging. In addition, as we previously demonstrated (Fichman et al. 2022), mutants deficient in ASCORBATE PEROXIDASE 1, that scavenges ROS, develop the ROS signal much faster than wild type, and mutants deficient in RESPIRATORY BURST OXIDASE HOMOLOG (RBOH) D/F develop the ROS signal much slower, as they produce less signaling ROS. The expression level of certain genes in specific tissues may therefore further influence the detection of the ROS signal. As we know very little about *Cuscuta* ROS metabolism, compared to *Arabidopsis*, the rates of signal detection may also be slower in *Cuscuta* due to similar reasons (i.e. dye uptake and ROS scavenging/production/transport). As we demonstrate the plant-to-plant signal transmission via 3 different dyes, with control experiments, and using RT-qPCR analyses (Figs. 1 and 2 and Supplementary Fig. S1), we are confident that our findings reveal the transmission of different plant-to-plant signals via a *Cuscuta* bridge, and that the different imaging intensities are simply a result of differences in dyes uptake and/or rates of ROS/calcium/membrane potential metabolism/signaling between the different tissues. In future studies, we would like to test the transfer of the plant-to-plant signal via a *Cuscuta* bridge using different *Cuscuta* mutants deficient in RBOHs or different ROS metabolism enzymes, as well as identify the source of ROS produced in *Cuscuta* and the 2 different plants during this response (other than RBOHs).

In a recent study, we demonstrated that under humid conditions plants growing in a community can transfer ROS and electric wave signals between each other, given that their leaves are physically touching each other (Szechyńska-Hebda et al. 2022). Here we extend our findings and report that individual plants that are connected through the parasitic plant *Cuscuta* can exchange systemic ROS, calcium, and membrane potential signals in response to local stresses such as wounding or high light stress. As the transmission of systemic stress signals between different plants connected by a *Cuscuta* bridge could activate acclimation mechanisms (Fig. 2, D and E) and contribute to the survival of these plants (and/or the entire community) during stress, it is possible that plant interactions with *Cuscuta* may provide certain benefits to plants, especially plants growing within a community. This possibility should be addressed in future studies as it may change the definition of *Cuscuta*/dodder from a parasitic plant to a partially mutualistic symbiotic plant.

## Supplementary Material

kiae339_Supplementary_Data

## Data Availability

The datasets used and/or analyzed during the current study are available from the corresponding author upon request.

## Supplementary data

The following materials are available in the online version of this article.

**Supplementary Figure S1.** ROS accumulation in donor, but not receiver plant, when the 2 *Cuscuta*-infected plants are not connected via a *Cuscuta* bridge.

**Supplementary Table S1.** List of primers used in this study.

**Supplementary Methods S1.** Planting materials and germination of *Arabidopsis*.

**Supplementary Methods S2.** Introduction of *Cuscuta* tissues.

**Supplementary Methods S3.** Facilitating parasitic connection between 2 *Arabidopsis* plants.

**Supplementary Methods S4.** Wounding and high light stress application.

**Supplementary Methods S5.** ROS, calcium, and membrane depolarization measurements.

**Supplementary Methods S6.** RNA extraction and RT-qPCR.

**Supplementary Methods S7.** Statistical analysis.

## Dive Curated Terms

The following phenotypic, genotypic, and functional terms are of significance to the work described in this paper:

PEROXIDASE CHEBI: CHEBI:8027

DIPHOSPHATE CHEBI: CHEBI:18361

NUCLEOSIDE CHEBI: CHEBI:33838

superoxide CHEBI: CHEBI:18421

CSD1 Gramene: AT1G08830
CSD1 Araport: AT1G08830

MYB30 Gramene: AT3G28910
MYB30 Araport: AT3G28910

NDPK2 Gramene: AT5G63310
NDPK2 Araport: AT5G63310

calcium CHEBI: CHEBI:29320

APX1 Gramene: AT1G07890
APX1 Araport: AT1G07890

## References

[kiae339-B1] Fichman Y, Mittler R. Integration of electric, calcium, reactive oxygen species and hydraulic signals during rapid systemic signaling in plants. Plant J. 2021:107(1):7–20. 10.1111/tpj.15360 34058040

[kiae339-B2] Fichman Y, Zandalinas SI, Peck S, Luan S, Mittler R. HPCA1 is required for systemic reactive oxygen species and calcium cell-to-cell signaling and plant acclimation to stress. Plant Cell. 2022:34(11):4453–4471. 10.1093/plcell/koac241 35929088 PMC9724777

[kiae339-B3] Grenzi M, Buratti S, Parmagnani AS, Abdel Aziz I, Bernacka-Wojcik I, Resentini F, Šimura J, Doccula FG, Alfieri A, Luoni L, et al Long-distance turgor pressure changes induce local activation of plant glutamate receptor-like channels. Curr Biol. 2023:33(6):1019–1035. 10.1016/j.cub.2023.01.042 36796359

[kiae339-B4] Hegenauer V, Fürst U, Kaiser B, Smoker M, Zipfel C, Felix G, Stahl M, Albert M. Detection of the plant parasite *Cuscuta reflexa* by a tomato cell surface receptor. Science. 2016:353(6298):478–481. 10.1126/science.aaf3919 27471302

[kiae339-B5] Hettenhausen C, Li J, Zhuang H, Sun H, Xu Y, Qi J, Zhang J, Lei Y, Qin Y, Sun G, et al Stem parasitic plant *Cuscuta australis* (dodder) transfers herbivory-induced signals among plants. Proc Natl Acad Sci U S A. 2017:114(32):E6703–E6709. 10.1073/pnas.1704536114 28739895 PMC5559024

[kiae339-B6] Hibberd JM, Dieter Jeschke W. Solute flux into parasitic plants. J Exp Bot. 2001:52(363):2043–2049. 10.1093/jexbot/52.363.2043 11559740

[kiae339-B7] Jhu M-Y, Sinha NR. *Cuscuta* species: model organisms for haustorium development in stem holoparasitic plants. Front Plant Sci. 2022:13:1086384. 10.3389/fpls.2022.1086384 36578337 PMC9792094

[kiae339-B8] Johnsen HR, Striberny B, Olsen S, Vidal-Melgosa S, Fangel JU, Willats WG, Rose JK, Krause K. Cell wall composition profiling of parasitic giant dodder (*Cuscuta reflexa*) and its hosts: a priori differences and induced changes. New Phytol. 2015:207(3):805–816. 10.1111/nph.13378 25808919

[kiae339-B9] Kollist H, Zandalinas SI, Sengupta S, Nuhkat M, Kangasjärvi J, Mittler R. Rapid responses to abiotic stress: priming the landscape for the signal transduction network. Trends Plant Sci. 2019:24(1):25–37. 10.1016/j.tplants.2018.10.003 30401516

[kiae339-B10] Liu N, Shen G, Xu Y, Liu H, Zhang J, Li S, Li J, Zhang C, Qi J, Wang L, et al Extensive inter-plant protein transfer between *Cuscuta* parasites and their host plants. Mol Plant. 2020:13(4):573–585. 10.1016/j.molp.2019.12.002 31812691

[kiae339-B11] Miller G, Schlauch K, Tam R, Cortes D, Torres MA, Shulaev V, Dangl JL, Mittler R. The plant NADPH oxidase RBOHD mediates rapid systemic signaling in response to diverse stimuli. Sci Signal. 2009:2(84):ra45. 10.1126/scisignal.2000448 19690331

[kiae339-B12] Mousavi SA, Chauvin A, Pascaud F, Kellenberger S, Farmer EE. GLUTAMATE RECEPTOR-LIKE genes mediate leaf-to-leaf wound signalling. Nature. 2013:500(7463):422–426. 10.1038/nature12478 23969459

[kiae339-B13] Nguyen CT, Kurenda A, Stolz S, Chételat A, Farmer EE. Identification of cell populations necessary for leaf-to-leaf electrical signaling in a wounded plant. Proc Natl Acad Sci U S A. 2018:115(40):10178–10183. 10.1073/pnas.1807049115 30228123 PMC6176584

[kiae339-B14] Slaby P, Körner M, Albert M. A cell wall-localized glycine-rich protein of dodder acts as pathogen-associated molecular pattern. Commun Integr Biol. 2021:14(1):111–114. 10.1080/19420889.2021.1918369 33995821 PMC8096328

[kiae339-B15] Suzuki N, Miller G, Salazar C, Mondal HA, Shulaev E, Cortes DF, Shuman JL, Luo X, Shah J, Schlauch K, et al Temporal–spatial interaction between reactive oxygen species and abscisic acid regulates rapid systemic acclimation in plants. Plant Cell. 2013:25(9):3553–3569. 10.1105/tpc.113.114595 24038652 PMC3809549

[kiae339-B16] Szechyńska-Hebda M, Lewandowska M, Witoń D, Fichman Y, Mittler R, Karpiński SM. Aboveground plant-to-plant electrical signaling mediates network acquired acclimation. Plant Cell. 2022:34(8):3047–3065. 10.1093/plcell/koac150 35595231 PMC9338792

[kiae339-B17] Toyota M, Spencer D, Sawai-Toyota S, Jiaqi W, Zhang T, Koo AJ, Howe GA, Gilroy S. Glutamate triggers long-distance, calcium-based plant defense signaling. Science. 2018:361(6407):1112–1115. 10.1126/science.aat7744 30213912

[kiae339-B18] Venkateshwaran M, Volkening JD, Sussman MR, Ané JM. Symbiosis and the social network of higher plants. Curr Opin Plant Biol. 2013:16(1):118–127. 10.1016/j.pbi.2012.11.007 23246268

[kiae339-B19] Zandalinas SI, Fichman Y, Mittler R. Vascular bundles mediate systemic reactive oxygen signaling during light stress. Plant Cell. 2020:32(11):3425–3435. 10.1105/tpc.20.00453 32938754 PMC7610290

[kiae339-B20] Zhuang H, Li J, Song J, Hettenhausen C, Schuman MC, Sun G, Zhang C, Li J, Song D, Wu J. Aphid (*Myzus persicae*) feeding on the parasitic plant dodder (*Cuscuta australis*) activates defense responses in both the parasite and soybean host. New Phytol. 2018:218(4):1586–1596. 10.1111/nph.15083 29575001

